# Managing a ventricular tachycardia storm: Looking beyond the horizon

**DOI:** 10.1002/joa3.70018

**Published:** 2025-02-13

**Authors:** Sanjai Pattu Valappil, Abhinav B. Anand, Prashanthan Sanders, Ramana Murugadass

**Affiliations:** ^1^ SRM Institute of Medical Sciences Chennai India; ^2^ Department of Cardiology Lokmanya Tilak Municipal General Hospital and Lokmanya Tilak Municipal Medical College Mumbai India; ^3^ Centre for Heart Rhythm Disorders, University of Adelaide, Royal Adelaide Hospital Adelaide Medical School Faculty of Health and Medical Sciences Adelaide Australia

**Keywords:** ablation, epicardial, multielectrode catheter, reentry, VT isthmus

## Abstract

The case highlights the possibility of nonischemic cardiomyopathy in patients with coronary artery disease and the complex nature of the isthmus with multiple entry and exit points. A combination of multiple strategies, that is, unipolar mapping, isochronal late activation mapping during sinus rhythm, and positioning of a multielectrode catheter at the putative isthmus during VT induction in the case of hemodynamically unstable VT, was used to achieve a successful outcome.

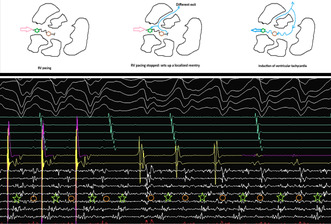

## 1 SPOTLIGHT

**FIGURE 1:**
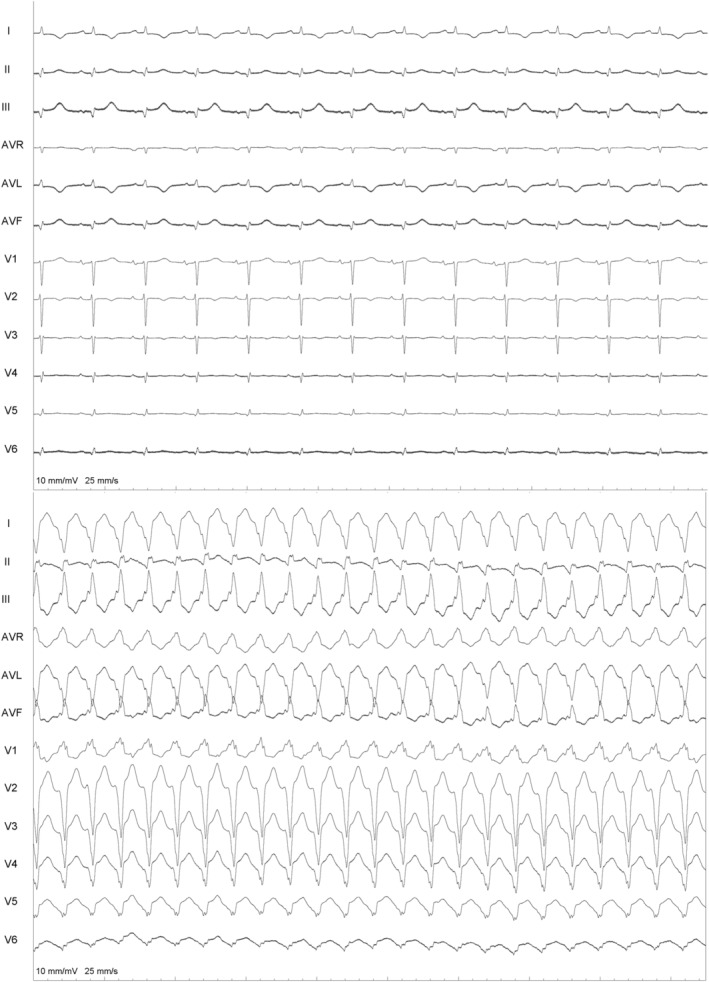
Twelve‐lead (12‐lead) electrocardiograms during sinus rhythm and ventricular tachycardia (upper and lower panel).

A 65‐year‐old gentleman, post recent coronary artery angioplasty and having moderate global left ventricular (LV) dysfunction, developed recurrent, drug‐resistant ventricular tachycardia (VT). The 12‐lead ECG during sinus rhythm and VT is shown in Figure 1. The VT showed a tall R wave in V1, Qs complexes in V2 –V5, Rs in V6, positivity in the inferior leads, and negativity in 1, aVL, indicating an apical, anterolateral exit. The patient underwent emergency DC cardioversion and was started on intravenous amiodarone (bolus and maintenance: 300 mg iv bolus over 10 min followed by 1 mg/min over 24 h). However, roughly 48 h after intravenous amiodarone (total loading: 2.5 g), patient developed recurrent (three episodes) episodes of symptomatic VT, which required electrical cardioversion in succession. The VT was resistant to intravenous lignocaine (1.5 mg/kg bolus followed by continued infusion 3 mg/min), stellate ganglion blockade, and additional boluses of amiodarone. The patient received a total of 11 DC cardioversions for recurrent symptomatic VT over a period of 48 h. A 2D echocardiography done at the bedside revealed generalized global hypokinesia (ejection fraction of 42%), more marked in the mid and apical anterior and anterolateral wall.

**FIGURE 2:**
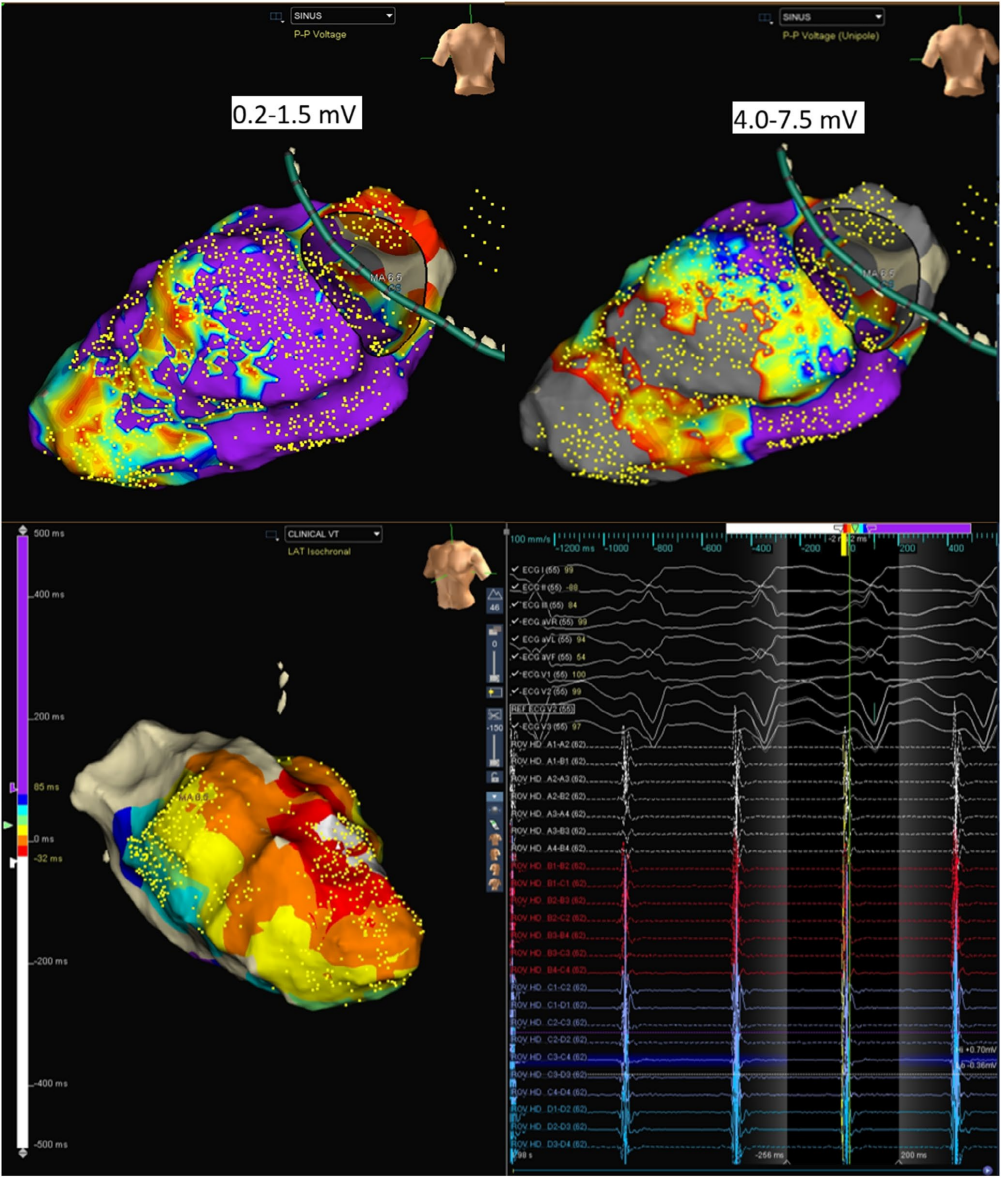
LV endocardial bipolar map (left) and unipolar map (right). Lower panel shows limited endocardial activation mapping because of hemodynamic instability, which shows a wide area of early activation without any mid‐diastolic activity.

Because of the incessant VT, the patient was taken up for catheter ablation using the 3D mapping Navx‐Ensite system. The patient was intubated and ventilated. The patient was in sinus rhythm at the start of the procedure (Figure 1). LV endocardial voltage mapping was performed first, which showed no noticeable scar in endocardial LV (0.2–1.5 mV). Tachycardia was then induced with ventricular extrastimuli, and focused mapping of the LV endocardium (because of hemodynamic instability) was carried out using the multielectrode mapping catheter. The LV activation map during VT showed a wide area of early activation in the mid‐apical anterolateral LV (Figure 2).

In view of the absence of diastolic potentials and a wide area of early activation, the critical isthmus was postulated to be located on the epicardial surface of the LV. The 12‐lead electrogram was reassessed. MDI taken at 300 mm speed was 0.67 (125/185). (Figure 2) The LV endocardial unipolar (4–7.5 mv) voltage map was next assessed; this revealed the presence of a large scar in the base to mid posterolateral and apical area (Figure 1). The base to mid lateral area corresponded to the wide area of early activation during endocardial mapping. Combined with the MDI information, the isthmus of the VT was highly suggestive to be epicardial.

**FIGURE 3:**
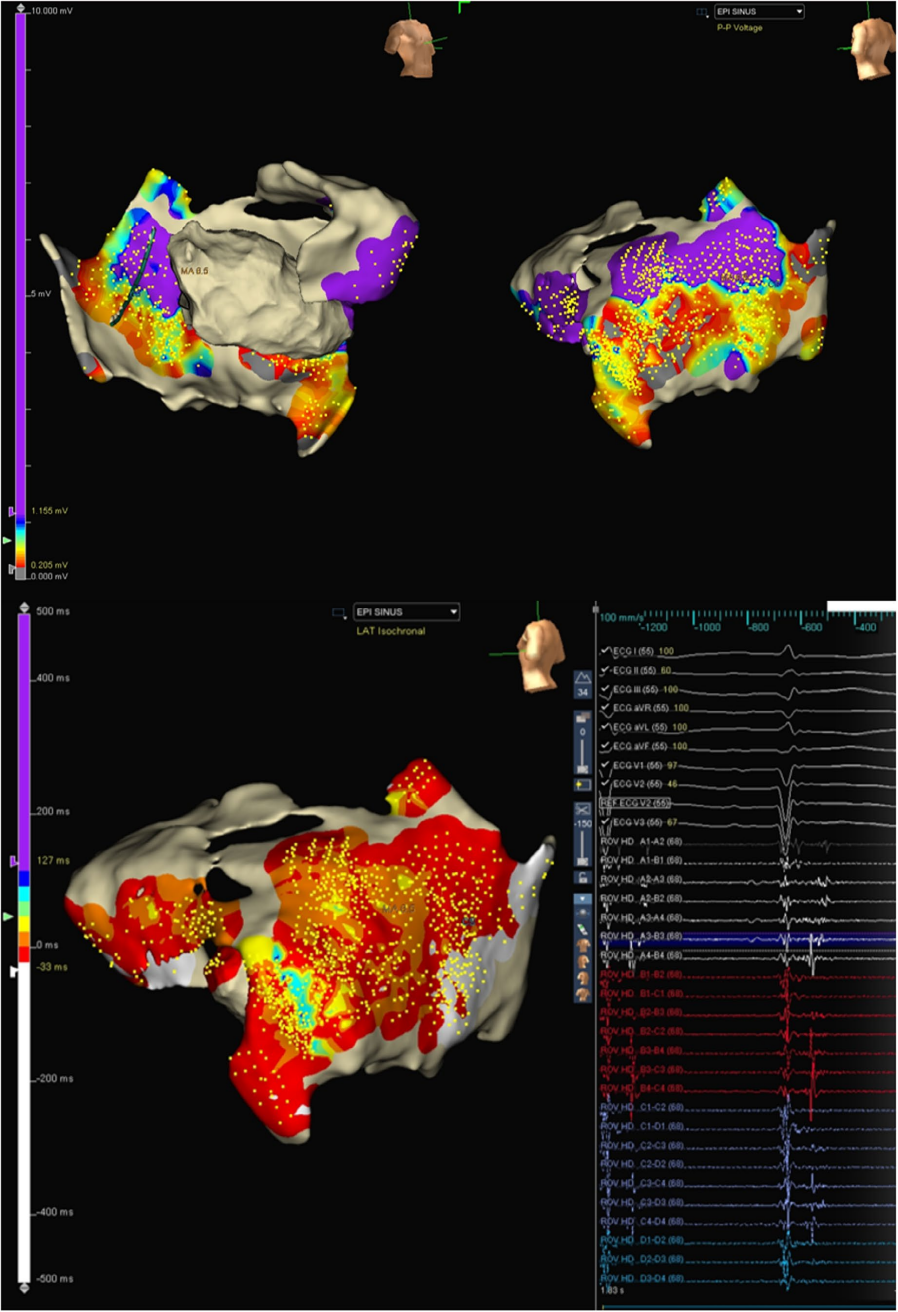
(A) Epicardial voltage map (upper panel). (B) Epicardial ILAM and presence of late potentials (lower panel).

**FIGURE 4:**
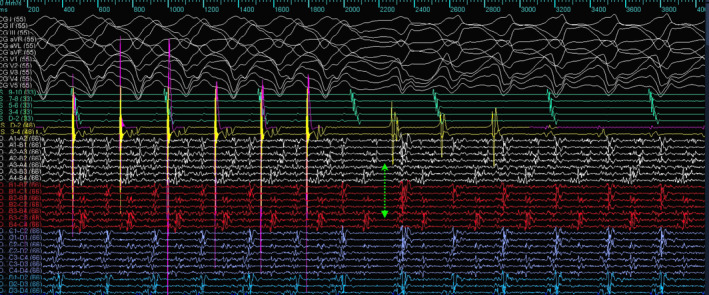
Induction of ventricular tachycardia from RV pacing. The multielectrode is placed in the region of epicardial ILAM. The green arrow marks the local conduction block in the isthmus inducing ventricular tachycardia; the first beat is narrower than the clinical VT and has another endocardial exit. The postulated mechanism of the tachycardia is explained in the next figure.

As the patient was on dual antiplatelets, the epicardium was accessed by a limited subxiphoid surgical epicardial window. The LV epicardial surface was mapped with the multielectrode catheter. The epicardial surface showed the presence of late potentials (LP). (Figure 3) Isochronal late activation mapping (ILAM), with the setting set to last deflection with sensitivity 0.1 mV, was performed with the sensitivity dynamically adjusted to make sure it sensed the last potential in the window appropriately, and re‐annotation was done as and when required during the procedure. To get the voltage map, the algorithm was switched from LAT to Peak to peak (P–P) and adjusted the sensitivity to 0.2–1.5 mV and 4.0–7.5 mV to get bipolar and unipolar voltage maps, respectively. ILAM showed the deceleration zone around the basal to mid posterolateral LV. The multielectrode was placed at the putative epicardial isthmus and VT was induced. (Figure 4).

**FIGURE 5:**
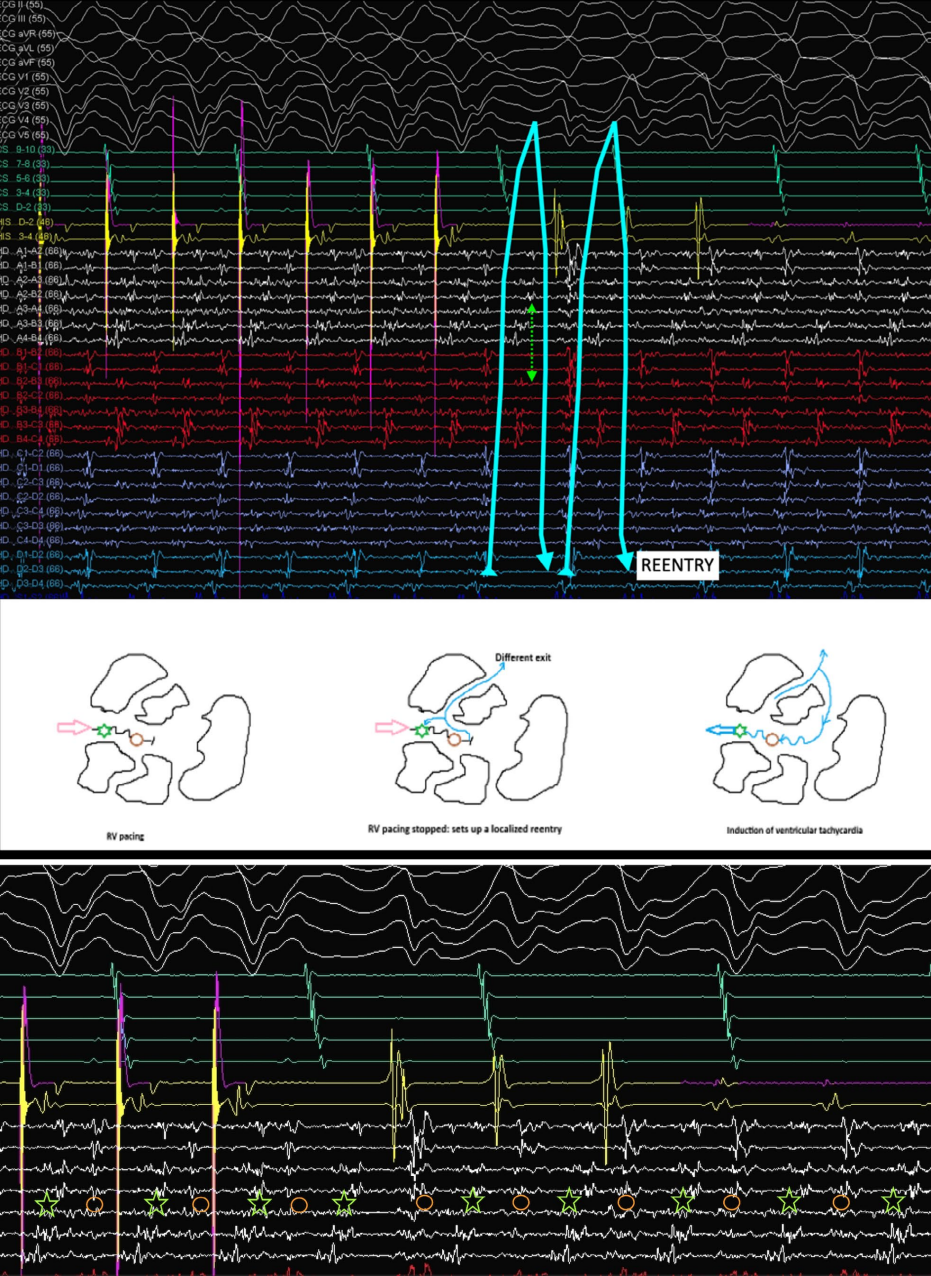
The upper panel shows localized conduction block followed by localized reentry. Magnified view of the local EGM and putative mechanism of induction of tachycardia (green asterisk and brown circle represent the reversal of activation during ongoing VT after induction, magnified from Figure 3).

The mechanism of VT can be elucidated from the tracing shown in Figure 4. The multielectrode catheter is placed in the area of isochronal crowding in the epicardium. During RV pacing, there are two distinct components on the multielectrode catheter, typically seen as an initial high‐frequency EGM followed by another low‐frequency signal in the channel A4–B4. When the RV pacing is stopped, there is induction of tachycardia that appears to initiate after a local conduction block in the isthmus, as evidenced by the lack of the second EGM on the A4–B4 channel. The first beat before tachycardia induction is different from the ensuing VT. Since this is narrower, this likely represents a different exit, which could be endocardial. With the initiation of the tachycardia, the multielectrode catheter channels show reversal of the electrogram components compared to the electrograms during the RV pacing, which are now spanning the entire diastole and represent a localized reentry. This phenomenon was reproducibly noticed during induction (Figure 5).

RF Ablation at this region at 40 W, 43°, 17 mL/min resulted in the termination of VT within 4 s. The ablation sets were given up to the inferior border zone of the epicardial scar and targeted the LP at the lateral epicardial scar border zone and anchored to the anterolateral mitral annulus. The patient was subjected to aggressive reinduction and no VTs were inducible.

The above case highlights the presence of nonischemic or dual cardiomyopathy in patients with coronary artery disease.^1^ Even though the patient was post‐angioplasty, the epicardial inflammation and scarring lead to the VT storm.

Our case is unusual for the fact that the endocardial bipolar voltage was completely normal. Bipolar voltage reflects the near‐field potentials, whereas intramural or epicardial potentials can be viewed on the unipolar channels.^2^ Endocardial unipolar mapping was thus utilized to identify an epicardial substrate since there were no endocardial bipolar voltage abnormalities. Since the unipolar potentials reflect far‐field tissue electrophysiological characteristics, changing to the unipolar settings allowed us to visualize the epicardial substrate, which revealed the presence of a posterolateral basal epicardial scar.

Since the patient had hemodynamically unstable VT, epicardial isochronal late activation in sinus rhythm was used to define the potential substrate.^3^ This corresponded to the area of wide early activation during VT in the limited endocardial mapping. The crowding of isochrones, as evidenced by the ILAM map, was located in the narrow corridor of channels within the wider unipolar scar. Comparing with the ILAM activation performed during sinus rhythm, the area of slow conduction accounted for the isthmus and the conduction barriers. Mapping of the epicardial ILAM thus allowed us to define the isthmus by placing the high‐density mapping catheter in the area of slow conduction and inducing the VT, as the VT in this case was not hemodynamically well tolerated.

The induction of the VT confirms reentry as the mechanism and highlights the complex nature of VT isthmuses characterized by multiple entry and exit points.^4^ Reentry was facilitated in this area because of the local conduction block within the critical epicardial isthmus, which was classically demonstrated by the EGMs in the high‐density mapping catheter. Multiple other VT morphologies were induced, which probably reflect the sharing of VT isthmuses in the areas with conduction slowing.

The combination of a structured electrophysiological approach in this case helped us to define a pure epicardial substrate in the patient. The LV activation map showed a wide area of early endocardial activation. The LV endocardial voltage map in unipolar map settings (4–7.5 mv) and critical reassessment of the ECG at 300 mm speed for MDI were done. The isochronal late activation map showed crowding of isochrones in the putative isthmus, and reproducible induction of tachycardia with local conduction block in the isthmus confirmed the epicardial substrate. In this case, not a single ablation was carried out endocardially.

Patient underwent successful implantable cardioverter defibrillator (ICD) implantation for secondary prevention. FDG PET CT/SPECT done after 3 months demonstrated increased uptake in the basal and mid apical inferolateral segments with perfusion defects consistent with inflammatory infiltrative cardiomyopathy. In view of the absence of accessible lymph nodes for biopsy and no arrhythmias post ablation, the patient was decided to be kept on clinical follow‐up and had no arrhythmia recurrence during the last 6 months.

## FUNDING INFORMATION

We confirm that we have not received any funding for the above study.

## CONFLICT OF INTEREST STATEMENT

The authors have no conflict of interest to declare. All coauthors meet the criteria for authorship and appropriate acknowledgments are made in the manuscript. In case of any errors in the data, the journal will be informed. We hereby transfer, assign, or otherwise convey all copyright ownership, including any and all rights incidental thereto, exclusively to the journal in the event that such work is published by the journal.

## ETHICS STATEMENT

This manuscript is not published anywhere else and is not for consideration for publication anywhere else.

## CONSENT

The informed consent from the patient has been obtained.
